# Hybrid clinical-managers in Kenyan hospitals: Navigating between professional, official and practical norms

**DOI:** 10.1108/JHOM-08-2017-0203

**Published:** 2019-03-28

**Authors:** Jacinta Nzinga, Gerry McGivern, Mike English

**Affiliations:** 1 Centre for Geographic Medicine Research Coast, Kilifi, Kenya; 2 Warwick Business School, University of Warwick, Coventry, UK

**Keywords:** Clinical managerial hybrids, Low–middle income countries, Practical norms, Professionals

## Abstract

**Purpose:**

The purpose of this paper is to explore the way “hybrid” clinical managers in Kenyan public hospitals interpret and enact hybrid clinical managerial roles in complex healthcare settings affected by professional, managerial and practical norms.

**Design/methodology/approach:**

The authors conducted a case study of two Kenyan district hospitals, involving repeated interviews with eight mid-level clinical managers complemented by interviews with 51 frontline workers and 6 senior managers, and 480 h of ethnographic field observations. The authors analysed and theorised data by combining inductive and deductive approaches in an iterative cycle.

**Findings:**

Kenyan hybrid clinical managers were unprepared for managerial roles and mostly reluctant to do them. Therefore, hybrids’ understandings and enactment of their roles was determined by strong professional norms, official hospital management norms (perceived to be dysfunctional and unsupportive) and local practical norms developed in response to this context. To navigate the tensions between managerial and clinical roles in the absence of management skills and effective structures, hybrids drew meaning from clinical roles, navigating tensions using prevailing routines and unofficial practical norms.

**Practical implications:**

Understanding hybrids’ interpretation and enactment of their roles is shaped by context and social norms and this is vital in determining the future development of health system’s leadership and governance. Thus, healthcare reforms or efforts aimed towards increasing compliance of public servants have little influence on behaviour of key actors because they fail to address or acknowledge the norms affecting behaviours in practice. The authors suggest that a key skill for clinical managers in managers in low- and middle-income country (LMIC) is learning how to read, navigate and when opportune use local practical norms to improve service delivery when possible and to help them operate in these new roles.

**Originality/value:**

The authors believe that this paper is the first to empirically examine and discuss hybrid clinical healthcare in the LMICs context. The authors make a novel theoretical contribution by describing the important role of practical norms in LMIC healthcare contexts, alongside managerial and professional norms, and ways in which these provide hybrids with considerable agency which has not been previously discussed in the relevant literature.

## Introduction

Clinical managerial “hybrids” – doctors, nurses or other health professionals in managerial roles – are playing a key role in improving healthcare systems in high-income countries by mediating between professional clinical and management practices and norms. Hybrids have both formal managerial accountability and informal accountability to professional peers, which may at times conflict. The way hybrids manage this tension has been found to affect strategic direction, management and outcomes in healthcare organisations and collaborative working with their own and other clinical professions (Fitzgerald and Sturt, 1992; Fitzgerald and Ferlie, 2000; Hoff, 2000; Llewellyn, 2001; Doolin, 2002; Forbes and Hallier, 2006; Fulop, 2012; Dickinson *et al.*, 2013; Fredrik Bååthe, 2013; McGivern *et al.*, 2015). However, research on hybrids has so far been limited to high-income country healthcare contexts, overlooking hybrids in low- and middle-income countries (LMICs). In this paper, we examine empirically and theoretically how hybrids understand and enact their clinical-manager roles in the LMIC context of Kenyan district hospitals.

Being a “professional”, particularly an elite professional like a doctor, provides prized status and often lucrative career opportunities, gained through long professional training, qualification and socialisation. Moreover, formative and ongoing professional training and socialisation greatly influences how professionals enact their roles. As a result, professional norms are entrenched within, and often dominate, healthcare systems. Historically, doctors risked their professional status by moving into managerial roles, particularly if they deviated too far from and challenged established professional norms (Mintzberg, 1989; Freidson, 2001). However, with increasing awareness of the need to provide healthcare with limited resources and following high profile professional scandals, which highlighted problems with professional modes of organising, managing healthcare became more legitimate. As hybrid clinical managerial roles also became more legitimate, those occupying these roles have been found to blend and hybridise health professionalism with management (Noordegraaf, 2007; McGivern *et al.*, 2015; Ham *et al.*, 2011).

Hybrids have been found to enact their roles in varying ways; some preserving traditional modes of professionalism and others challenging professionals and aligning them with managerial contemporary healthcare settings (Forbes and Hallier, 2006; McGivern *et al.*, 2015). A key factor affecting the way hybrids enact their roles may be whether they are in hybrid roles “reluctantly” (Forbes and Hallier, 2006), “incidentally” or “willingly” (McGivern *et al.*, 2015). McGivern *et al.* (2015) show how “incidental hybrids”, who took managerial roles out of a sense of obligation, enact them in a way that protects and preserves professional norms. “Willing hybrids”, who are interested in managing and therefore willingly took managerial roles, enact hybrid roles in ways that challenge traditional professional norms, blending them with managerial norms. However, research on clinical management and leadership has, so far, been limited to high-income country healthcare contexts, with little research in LMICs (Daire and Gilson, 2014). So, do we see similar patterns among hybrids in LMIC health systems?

In Kenya, county (formerly district ) hospitals are an important part of the health system, delivering essential healthcare services to more than 80 per cent of the population and employing over half of all healthcare staff (Couper *et al.*, 2002). However, the functioning of these hospitals is complex. The formal and informal rules regulating them are poorly understood, characterised by complex hierarchies, often with poor alignment of responsibility, poor accountability and strict role boundaries between professional groups (Nzinga *et al.*, 2018). Furthermore, perhaps consequently, their performance is often inadequate, characterised by failure to implement international and national best practice (Nzinga *et al.*, 2009; English *et al.*, 2011). There is therefore interest in understanding the way such contexts affect health workers, particularly clinical managers, and their efforts to improve services.

In many such settings management functions are undertaken by clinicians without management training (O’Neil, 2008). Oftentimes, clinicians take on senior management roles with the support of just a few administrators with basic management, administrative and accounting training specific to routine health service policies and procedures (Soong *et al.*, 2010). These senior clinical managers typically also co-opt professionals in charge of nursing, pharmacy and allied health services in hospitals and medical and nursing department heads, who are involved in planning and coordinating services in their departments. These middle-level hybrids thereby bridge the gap between management and clinicians and senior management and frontline workers in hospitals (Nzinga *et al.*, 2013; Tetui *et al.*, 2016).

Furthermore, clinical managers in African public healthcare also need to navigate institutionalized but informal “practical norms”, which affect behaviour in such settings. Practical norms are defined as “the various informal, de facto, tacit or latent norms that underlie the practices of actors, which diverge from the official norms (or social norms)” (De Sardan, 2015, p. 26). Practical norms are different from official (managerial), professional norms or traditional cultural and social norms, in that they explain informal and implicit practices, which deviate from official policy but are nonetheless widespread, accepted and regulate most African public servants’ behaviours. Practical norms exist in the absence of the law, or where law cannot be effectively enforced, and while not made explicit or officially taught are learned and then implicitly enacted in the practices of many public servants (De Herdt and De Sardan, 2015).

Practical norms often involve deliberate pragmatic infringements of rules, which provide a way of evading official rules, creating space for gaming, and providing agency by a wider repertoire of actions possible. As De Sardan (2015, p. 39) notes: “the actors’ ‘game’ or margin for manoeuvre consists in navigating between official, social and practical norms, i.e. moving closer to some or others depending on the context, the personal options available to them or the nature of interactions involved”. Practical norms may produce adaptive change as they mutate into quasi-official norms tailored to and justified by constraints or limited resources in particular contexts.

Thus, in the absence of formalized and enforceable official (managerial) norms, we suggest that hybrid roles may be affected by practical norms. As practical norms influence LMIC health systems, alongside clinical professionalism and managerialism, supplying alternative ways of enacting roles, hybrids in LMICs, must navigate and reconcile not only professional and managerial expectations but also practical norms. We adopt this theoretical framing to explain the way hybrids in Kenyan county hospitals interpret and enact their roles. Yet, De Herdt and De Sardan (2015) note that while some practical norms operate openly, other practical norms may be hidden, social ethnographic research is needed to uncover and explain them. Accordingly, drawing on ethnographic research methods, we address the following research question:

RQ1. How do health professionals interpret and enact hybrid clinical managerial roles in LMIC healthcare settings affected by professional, managerial and practical norms?

## Study setting

Kenya’s National Health Sector Strategic Plan (NHSSP) 1999–2004, and later NHSSP-II (2005–2010) set out activities, costs and targets for healthcare improvement. Both policies recognised that training for managers throughout the health system would be necessary. Consequently, numerous leadership and management courses ran for health managers. Yet, in 2008, a national survey documenting health managers’ leadership and management capacity within the public and the private health sectors revealed major gaps. While more than 90 per cent of all health managers surveyed viewed quality management as important, 61 per cent said that they felt inadequately prepared for their role as health managers (MSH *et al.*, 2008). Part of the problem is that the training provided largely focussed on technical competencies rather than the practical challenges managers face in Kenyan hospitals. Indeed, there remains little understanding of these challenges or how to support healthcare managers to improve health service delivery.

Within most Kenyan hospitals, there are broadly two levels of managers; senior managers and mid-level managers. Senior managers include a medical superintendent (in effect, a medically trained hospital chief executive officer), a hospital chief nurse (hospital matron) and a hospital administrator (without a clinical background but with basic management, administrative and accounting training specific to routine health service policies and procedures) (O’Neil, 2008). These senior managers lead a hospital management team, which holds administrative power and typically co-opt professionals in charge of clinical departments into clinical managerial roles.

In this paper, we focus on departmental managers who head clinical services in Kenyan public hospitals (medicine, paediatrics, medicine, obstetrics and gynaecology and surgery). Unlike senior managers, these managers maintain their clinical roles alongside their managerial role while having responsibility for translating management objectives into practice (Nzinga *et al.*, 2013). However, as noted above, most Kenyan clinical managers lack managerial training (The Kenya Health and Leadership Congress, TMOH, 2015).

Typically, there is one consultant physician and one nurse manager in each hospital department. Physicians heading departments commonly have a higher degree in an appropriate medical specialty but as few as five years’ work experience (including three specialist years training). Nurses leading departments tend to have more work experience but few have higher training in a specific clinical specialty (such as paediatric or surgical nursing) (Nzinga *et al.*, 2013).

Nationally, an informal but institutionalized practical norm dictates that medical consultants in Kenyan county hospitals should automatically be considered as heads of clinical departments. With inadequate clinical consultant numbers countrywide, most county hospitals have only one consultant per specialty. These consultants therefore have no choice but to assume clinical managerial roles. Conversely, nurse managers are appointed following seniority criteria, a managerial position represents career development and advancement for nurses.

However, the complexities these Kenyan hybrid clinical departmental managers face are poorly understood, and examining and theorising their experiences provides the opportunity to extent the hybrids literature into LMIC contexts.

## Research design and methodology

We adopted a qualitative case study methodology, which is useful for exploring and generating explanation of complex social phenomena situated in context (Yin, 2013). The case study examined hybrids at three levels of analysis: individual hybrid professionals (their personal experiences, knowledge and practice); hybrids collectively (shared/collective meaning making and relationships) and the organisation (hospital) context in which hybrids worked.

We purposively selected two county public hospitals (see Table I) based on several considerations: both hospitals were designated as county hospitals; the hospitals faced varied challenges, were of different sizes, had different levels of human resources and contained managers with different levels of experience; we specifically selected hospitals where middle-level clinical managers had undergone some leadership and management training to see how this affected the way they enacted their professional/managerial roles; finally, the hospitals had to be open to being researched, as the research involved multiple visits and long interviews. Selecting hospitals in this way meant that we captured rich data about different kinds of hospitals, although this also means that they are not necessarily representative of all county hospitals in Kenya.

We conducted semi-structured interviews with eight clinical managers who were the focus of this study, asking them to reflect on entering a clinical or nursing career, then taking and enacting clinical managerial roles, and their perceptions and experiences being a hybrid. We then conducted focus group discussions with 51 frontline workers and six senior managers, exploring other clinicians’ and nurses’ perceptions of how the hybrids enacted their roles. We also engaged in non-participant observations, shadowing eight hybrids during routine practice. During this shadowing we had informal conversations with study participants, which were useful in informing the data analysis process. We kept detailed notes on emerging codes, which we used to inform subsequent interviews and interpretation of data collected. Thus, data analysis was conducted alongside data collection, which enabled an iterative theorisation process (Gioia *et al.*, 2013; Corbin and Strauss, 2014).

We coded data using NVIVO 10 software. Our iterative coding and theorisation drew upon the “Gioia method” (Gioia *et al.*, 2013), involving induction and deduction. Coding was initially framed and sensitised by pre-existing theory about hybrids. We then engaged in line-by-line coding of our interview and observational data, looking for key phrases and concepts (e.g. how hybrids were appointed to their roles or hybrid job descriptions). Next, we grouped related concepts into an all-inclusive category (such as transition into a hybrid role). Then, we made connections between categories, considered prior theory and literature, and explored the role of context on hybrids’ transitioning and role enactment.

We went through iterative cycles comparing data and theory. Reviewing literature on hybrids, we found little explanation of the ambiguous context and norms that Kenyan hybrids described facing. We therefore turned to De Herdt and De Sardan’s (2015) ideas about practical norms in African public services as a potentially useful theoretical frame explaining how Kenyan hybrids enacted their roles. We kept theoretical memos during data coding, which helped us keep track and make sense of developing ideas and concepts and form a mid-range theory that explained our findings. Thus, our theorisation involved a continuous process of reflection and comparison between empirical findings, emerging codes and theory to develop a theoretical explanation (Eisenhardt, 1989; Langley and Abdallah, 2011; Gioia *et al.*, 2013; Corbin and Strauss, 2014) of how hybrids took on, understood and enacted their roles.

## Results

Before we present our empirical findings, we comment on the contextual influences of the case study hospitals. Although our selection criteria for the two case hospitals assumed that there would be differences between them, based on contextual differences (e.g. geographical setting, work volume, infrastructural differences, clinical managers, etc.), we found that hybrids’ experienced similar patterns in both hospitals in relation to: recruitment and transitioning into hybrid roles; negotiating tensions between clinical and managerial aspects of their roles; and the way practical norms affected and provided a resource in navigating role conflicts. We present these themes below.

### Transition into hybrid roles

In both hospitals, hybrids described either being “thrown” into the role or being implicitly aware (without this being formally discussed) that they would have to step into the role, as there was no one else who could take it:

If you are the only surgeon then automatically you become the HOD [head of department] […] there’s not really strict criteria […] you’ll find the senior most in that department would be the in-charge

(Medical Superintendent, Hospital B).

New hybrids were commonly unprepared and untrained for administrative or management issues, having only received clinical training:

We are not even trained to be administrators […] you are suddenly dumped in that office […] you start learning from scratch. We are not real managers […] we didn’t go to school anywhere to learn managerial skills

(Obstetrics/Gynaecology Consultant, Hospital A).

Thus, most hybrids resorted to using their clinical backgrounds to make sense of their new roles. As one consultant paediatrician (Hospital B) noted:

As a pediatrician, my view of the work [being head of department] is that I am supposed to work in the ward, managing the children, working in the clinics and managing students.

Oftentimes, hybrids were left to figure out management responsibilities, “learning” and “creating roles as they go along”. Furthermore, because clinical work was standardized and predictable compared to management, hybrids selected their managerial responsibilities based on what was appropriate for them primarily as clinicians, often distancing themselves from more managerial aspects of their roles:

[Senior management] used to have Hospital Management Team meetings on Wednesday […] I’d feel like we spend the whole day there […] So, what I used to do is I would go to the New Born Unit and do a [ward] round and just come up and catch up with them at wherever point […] I don’t really see the value of these [management] meetings

(Paediatric Consultant, Hospital B).

Hybrids were subject to informal role expectations from senior managers and frontline clinical workers but both hospitals lacked job descriptions or formal guidance for clinical managerial responsibilities. This gave hybrids autonomy to choose what to do (or not do) within their managerial role.

Senior managers’ expectations of hybrids, based on generic national schemes of service for health workers, corporate hospital values and generalised professional task expectations, were also unclear:

There isn’t really a job description […] a medical officer’s role is general, so whatever you find at your [work] station it is within your capacity and you should be able to do it. So, what I got was an orientation of the place, what goes on every day, and then the rest is what is expected of me as the doctor. So, basically, I help in doing most of the procedures in the department; I help in training the interns, doing ward rounds, running clinics and we run the Maternal Child Health clinics. And doing most of the procedures, at least most minor surgical procedures and a few majors like caesarean sections

(Obstetrics/Gynaecology Medical Officer, Hospital B).

A recurring complaint among hybrids was lack of recognition (whether in kind or in terms of compensation or payment) for their managerial role, especially because many felt it took a considerable amount of their time:

In this hospital you are called an “in charge”, you are called a “deputy in charge” […] [but] there is no official communication, as in if you go to my file there is nothing that indicates that I’m an in charge or a deputy in charge. At least it’s my hope that the hospital recognizes such positions through promotion

(Paediatric Nurse Manager, Hospital A).

Being a head of department doesn’t come with anything in your pocket. It just gives you more work and more responsibilities but there is nothing you would benefit from as a person

(Medical Superintendent, Hospital B).

Some nurses were more willing to take manager roles, which we noted earlier were seen in terms of career progression for them. One nurses’ description of her transition was feeling:

A bit excited, because for one, when you become a nurse manager at least you had a […] I want to call it a special something […] because your views are at least taken with weight

(Nurse Manager, Hospital A).

Doctors were usually reluctant to take on managerial roles, as one recounted:

My core training is Obstetrics/gynecology, you are pushing me so hard with management. Do I leave the patients? […] I feel no, if I have to balance it any time I’ll take on my patients and leave out the management

(Obstetrics/Gynaecology Consultant, Hospital A).

Therefore, professional norms, official hospital management norms (usually perceived to be dysfunctional and unsupportive), and local practical norms developed in response to this context all affected hybrids’ understandings and enactment of their roles.

### Negotiating the duality of hybrid roles

Hybrids in this study had to learn to negotiate their clinical and managerial roles. Reflecting McGivern *et al.*’s (2015) and Forbes and Hallier (2006) typology of hybrids, we found “willing” and “reluctant” or “incidental” clinical managerial hybrids. However, the ambiguous and changing healthcare context and practical norms Kenyan hybrids worked with meant that there were also some “ambivalent hybrids”, who sometimes embraced their managerial role and at other times distanced themselves from it. Of the eight hybrids we analysed, we categorised four as reluctant, two as willing and two as ambivalent hybrids.

Reluctant hybrids reported “being pushed” into the role or “taking one for the team”. They exhibited no desire to learn how to manage, as one reluctant hybrid nurse manager noted:

It is a big responsibility and, in your heart, you don’t accept it but you have to take it. And again, if you refuse that, you don’t take the responsibility, you will not have assisted the team. So, you just take it

(Maternity Nurse Manager, Hospital A).

Reluctant hybrids feared additional responsibility and considered managerial duties an added burden on top of their already demanding (and more important) clinical roles:

You find that you’ve carried a heavy burden [taking on a managerial role] because you are there, you are working like any other nurse and also, you are there as the administrator. Also, the issue of supervision; like now I have interns who I’m working with as their in charge. So, I’m supposed to supervise, orientate and also give them the technical advice […] you might want to give the patient holistic care but you find it comes with a lot of other responsibilities, you see. Those kinds of the things give us a lot of tension

(Paediatric Nurse, Hospital B).

Furthermore, organisational histories influenced the emergence of reluctant hybrids. For example, most reluctant hybrids described difficult relationships with senior managers and limited control over resource and budgetary allocations for their departments. They therefore resented being in clinical management roles without the support or authority they required:

Most of my colleagues are demoralized; they are trying to adjust to the change [becoming clinical managers] […] it’s because they are not directly involved in the decision making. And that decision making is coming from above; they [senior managers] give us orders […] you will be called to be told ‘this and this is happening in your department, so and so is going for a training’ [heavy sigh]. First you have wasted my time, I am supposed to be off to doing my things, you tell me to come to just give me an information on what you are doing, I am not hands on. So that one really puts me down

(Paediatric Nurse Manager, Hospital B).

The predominantly reluctant responses of hybrids to their roles mirrors early research on hybrid doctors in the UK who were initially reluctant to take on managerial roles and responsibilities (Fitzgerald and Sturt, 1992; Fitzgerald, 1994).

By contrast, Kenyan willing hybrid stalked about receiving support and encouragement from senior managers during their transition into the hybrid role and noted the importance of having mentors and role models who had inspired them:

I learnt a lot of the administrative things […] when I became a manager in this hospital. I was just learning from the Medical Superintendent, who has been here for a longer time […] we used to blame the admin that they are not doing things right but we realized actually the mistake was ours. I realized actually I have a great role to play in the procurement chain, even in terms of receiving when the things are brought […] I should be able to look at it and say this is what I needed or this is not what I wanted. So, I think she [Med Supt] has helped me in that way

(Paediatric Consultant, Hospital A).

This finding resonates with research showing that “willing” hybrids have been shown by earlier mentors and role models how to enact organisational changes and implement improvements in ways they perceive to benefit healthcare staff and patients (Goodall, 2011; Ferlie *et al.*, 2012; McGivern *et al.*, 2015).

Indeed, willing hybrids saw the transition into a management role as an opportunity to be innovative, role model desirable behaviour for colleagues and juniors, and blend their clinical and managerial practices:

Initially I was like, I’ll just continue doing what I am but then I think I have felt the challenge […] I’ve realized there’s still more I can do. And then it has also exposed me to realize the problems even my juniors are facing because in the process of managing you also get exposure. So being an in charge, I was looking mainly at individual cases but now I look at it wholly […] I don’t only look at being an in charge I’m also doing research how to be a good leader, I took it wholly and accepted it

(Maternity Nurse manager, Hospital B).

Willing hybrids described personal characteristics and behaviours that had prepared them for a leadership or managerial role. For example, they talked about “being a performer, setting examples for others, being strict on rules, solving issues, etc.” For one “willing” nurse manager, her hybrid role appealed because it offered “authority” to influence behaviours in a way she was unable to do as a regular nurse:

At least with being an in-charge, I can change situations, like a wrong I have seen somewhere. With the authority I have as an in charge, I am able to change that. Or maybe when there is a mistake somewhere by a junior and it is affecting the management of the children, with the authority I have they have to respect the office of the nursing officer in charge of the department […] if I wasn’t an I charge, I wouldn’t be able to correct anyone

(Nursing Manager, Hospital A).

Some hybrids remained “ambivalent” about their clinical management roles, torn between the demands of both clinical and managerial responsibilities. These “ambivalent hybrids”, who were both nurse managers in our study, moved between identifying and dis-identifying with their hybrid role, depending on the prevailing contexts, as illustrated in the interview extract below:

It [taking on the managerial role] depends […] because there are times you get a lot of discouragement. You just want to write a resignation letter and ask to be relieved of being an ‘in charge’ [hybrid nurse manager role]. But, then again, I ask if I don’t take up the responsibility then who will? Okay I can say at first I didn’t expect it […] then it became exciting but now on the job there are challenges, so I don’t really know

(Paediatric Nurse Manager, Hospital B).

Ambivalent hybrids commented on poor organisational support. Consequently, they distanced themselves from their hybrid role where they could not enact it effectively, while at other times using it to exert authority, drawing on ambiguous practical norms to serve their personal agendas. Hence, we again show how Kenyan hybrids drew on dominant professional norms, often dysfunctional official managerial norms and informal local practical norms to enact their roles.

### Navigating role conflicts

Hybrids in our study faced a number of other challenges, which included lack of resources, overwhelming managerial responsibilities, poor accountability and managing the conflicting expectations of senior managers, peers and juniors. Senior management expected clinical department managers to be involved in resource planning and budgeting, while they had to compete for limited resources with colleagues from other departments. Furthermore, junior staff also expected them to exude expert knowledge and relational skills as described below:

There is potentially so much because you are supposed to do to coordinate the services. You must be part of the HMT [hospital management team] and you will be appointed to head a committee […] if it is the training committee, to look at the training needs, issues to do with trainings and quality improvements, etc. You should be part of the team [HMT] when planning and managing how to use available resources. Sometimes the money is not enough, so even if my department collects money we have to give some departments some money to run because some of them don’t get money and then we wait to raise our needs in the next management meeting

(Obstetrics/Gynaecology Consultant, Hospital A).

They [Frontline staff] expect to have […] affection for colleagues. Because you might be too harsh following the rules and then you don’t bother with the personal issues which also affect work. So, for the sake of improving their morale, you should also have that soft part of knowing really what is happening with so and so. So personal concern is also an issue as a manager you should know and have

(Maternity Nurse Manager, Hospital B).

Furthermore, in the hospitals we studied, as in most Kenyan county hospitals, there were complex hierarchies with unclear lines of accountability, strong professional allegiances and varying competing interests, which posed challenges for hybrids. For example, medical consultants determined how many hours they spent in their hospital, often only spending the minimum 12 h per week necessary to meet senior managers’ low expectations. Consultants getting away with doing the bare minimum, seemed to be a local practical norm, as illustrated below:

During my internship, he (medical consultant) used to show up to the wards only once a week […] for the major rounds. Even now, if you call a consultant, he gives you instructions on the phone or he comes once a week to the wards, goes through the patient’s files and if there is no issue he goes away

(Obstetrics/Gynaecology Medical Officer, Hospital A).

In the absence of management education and skill, hybrids relied heavily on their understanding of hospital contexts to manage the day-to-day issues in their department. Often hybrids used prevailing practical norms to legitimate their role. For example, most hospital departments lacked clear goals and staff orientations. Without clear work targets or bureaucratic procedures, hybrids were often unaccountable for their conduct, both upwards and downwards, so only performed role expectations when senior managers were watching:

Sitting in meetings as management heads of department, you budget on things that will never be acted upon […] Sometimes you realize some minutes are the same ones we have four months ago. And the response is “no funds”. “Why don’t you fix this and this?” “We don’t have this”. “Anything can be done about that?” “No”. “Next minute” […] Useless! I’d take up management more keenly if I thought my views will be respected, and they’ll be factored into the major decisions that are being made for the facility

(Obstetrics/Gynaecology Consultant, Hospital B).

I try as much as possible that by the close of the day there’s nothing pending. But you wouldn’t actually have […] specific goals

(Obstetrics/Gynaecology Consultant, Hospital A).

From our observations, work in both hospitals was shaped by practical norms. For instance, hybrids were subject to only (what interviewees saw as) ritualistic annual staff appraisals. As one noted:

Annual performance appraisals are like a ritual […] people do not look at it as something that can help them, because there is nobody who has been promoted or given extra pay for working hard. And everybody scores a hundred [%] because nobody wants to really do the right thing. Nobody wants to tell the person “you are doing something wrong here”

(Medical Superintendent, Hospital A).

Mandated official managerial processes (such as performance appraisals and routine documentation) were implemented in a way that depended on localised understanding and practical norms, which often focussed on shifting blame and using processes protectively, as illustrated below:

The game of documentation, we first took it from paediatrics whereby if an IV [Intra-venous] line has dislodged into a body tissue a nurse documents, “the CO [clinical officer] intern informed at 4 pm to run the tissue.” After one hour it’s written again […] after two hours “[…] and it can go on even for 48 hours, she’s still writing, she’s still writing. And you know why? It’s a game of now checking who is not working and why? […] Everyone is trying to clean up himself or herself”

(Clinical Officer Intern, Paediatric Rotation, Hospital B).

Finally, even though most hybrids we studied were unenthusiastic about their clinical management role, and complained about lack of preparation and training for the role, they expressed little interest in undertaking management education. This is because management work is seen to get in the way of what is perceived to be more important clinical work. Conversely, despite unstandardised work practices, poor clinical accountability, heavy workloads and shortages of resources and staff; few hybrids thought about using management to improve the provision of care, while most hybrids continued to show enthusiasm for clinical roles:

During my major ward round, I will see every baby in that ward. Whether they are 60 or how many I’ll try and I’ll see the 60. In between when I come for reviews then that time then I will see maybe the very sick babies. It means I spend every day here. But with management the stress is too much because there are a lot of issues and you still get blamed for things. I think the most thankless thing anybody could ever have to do is being a manager - it takes you completely away from the clinical. You see like Dr X is in the office he cannot take part in clinical duties, he is more involved in those other administrative duties […] I think if I didn’t see these babies I would die

(Paediatric Consultant, Hospital B).

In summary, the tensions between managerial and clinical roles were difficult for hybrid managers. In the absence of management skills and effective structures, they drew meaning from clinical roles, navigating tensions using unofficial practical norms.

## Discussion

We explain how hybrid clinical managers in Kenyan county hospitals interpreted and enacted their roles during routine clinical practice. Research on hybrids in Europe and North America has described how they navigate between competing professional and managerial norms (Hoff, 2000; Llewellyn, 2001; Doolin, 2002; Fulop, 2012; Dickinson *et al.*, 2013; McGivern *et al.*, 2015). We used the concept of “practical norms” (de Sardan, De Herdt and De Sardan, 2015) to show the added complexity that Kenyan hybrids face navigating three competing norms.

Moreover, research in high-income health contexts has identified different types of hybrids (willing and incidental/reluctant) (Forbes and Hallier, 2006; McGivern *et al.*, 2015), who took on clinical managerial roles for contrasting reasons and enact them in different ways. We too found willing and reluctant/incidental hybrids and, like willing hybrids in the UK (McGivern *et al.*, 2015), Kenyan willing hybrids comment on having managerial mentors who had encouraged them and demonstrated how these roles could be enacted effectively and indeed these Kenyan “willing” hybrids saw their roles as a means to making improvements. However, we also found a third type; the “ambivalent hybrid”. The ambiguity and variability of the Kenyan health context and practical norms meant that ambivalent hybrids both embraced and “distanced” (Goffman, 1961) themselves from their clinical managerial roles, depending on whether the role enabled incumbents to make a difference or not. Thus, ambivalent hybrids avoided being associated with management in situations likely to undermine their professional legitimacy but used the same managerial roles to increase their status, authority and advance their personal interests or agendas where it was useful.

Although the introduction of managerialism was intended to improve public-sector efficiency, effectiveness, standardisation and accountability (Bangura and Larbi, 2006) in the Kenyan context of our study, management appeared to be characterised more by chaos and confusion. Consequently, few clinicians showed interest or enthusiasm for taking on a hybrid role or using management to improve healthcare. Organisational support (at hospital and county level) to recruit hybrids and help them transitioning from clinician into hybrid clinical-manager was also lacking. Yet, clinicians often felt compelled to take on hybrid roles, which they knew were not valued or possible to enact well. Thus, Kenyan hybrids in our study commonly rejected organisational prescriptions, which were perceived to clash with their clinical role, and instead drew on clinical norms they and their professional colleagues understood and valued more, to enact their hybrid roles.

Hybrids in this study also used practical norms to reconcile the inconsistencies between lived experiences, professional, social norms and official managerial prescriptions often perceived as impractical and un-implementable, as also found elsewhere (Anders and Makene, 2018). Thus, hybrids’ exhibited agency by navigating between official, professional and practical norms to survive working in chaotic and often dysfunctional hospital environments. This reflects De Sardan’s (2015, p. 39) description of “moving closer to some or others depending on the context, the personal options available to them or the nature of the interactions involved […] having a repertoire of available guides to action […] practical norms are agency friendly”.

Research has shown that discretion and control are of crucial importance to clinical managers in LMIC healthcare settings (Doherty, 2013). Clinical managers are reluctant to take on managerial roles or participate in resource decision making processes in LMICs where they are not given autonomy to make decisions (Barasa *et al.*, 2017). Yet Nyikuri *et al.* (2017) showed how clinical managers coped with changes following devolution in the Kenyan health system by reducing some managerial activities, re-organising work routines and building on experience and relationships. From our findings, hybrids, particularly ambivalent hybrids, appeared to “play the system”; sometimes adhering to managerial objectives but more often taking advantage of local practical norms (the “local professional culture”, informal habits, tricks and strategies, and related ambiguity) to their own advantage.

Hybrids’ practical norms also included avoidance of challenging poor behaviour and practices, e.g., failing to confront peers over poor clinical practice or unauthorised absences, again as found in other studies of Kenyan healthcare managers (Brown, 2016). These behaviours seem to have become part of practical norm relating to Kenyan professionalism and suggesting professional allegiance (Blaise and Kegels, 2004; Nzinga *et al.*, 2009). Another practical norm appeared to be using officially instituted formal routines and paperwork, such as routine documentation during service delivery, which were officially meant to encourage learning from errors, to shift blame for malpractice, as found elsewhere (Nyikuri *et al.*, 2015; Litorpa *et al.*, 2015). Similarly, annual performance appraisal processes were enacted in a ritualistic manner devoid of content. This ambiguity and informality in practical norms relating to the enactment of managerial processes, provided hybrids with autonomy, flexibility and agency to enact their roles in ways that suited them.

The above examples illustrate what De Sardan (2015) refers to as the notion of “normative pluralism”, where official, professional and practical norms co-exist and actors have agency in relation to which and to what extent they comply with of these competing norms. Accordingly, we suggest that Kenyan hybrids have agency than hybrids in high-income counties do not have, because Kenyan hybrids have more (managerial, professional and practical) norms to draw upon than hybrids in high-income counties caught between managerial and professional norms (Noordegraaf, 2007; McGivern *et al.*, 2015; Ham *et al.*, 2011; Quinn and Perelli, 2016). However, at the same time, Kenyan hybrids may have less agency to make changes to the healthcare organisations they work in due to the dysfunctional managerial systems they appear to work within and because practical norms also undermine them. Further research regarding the sustainability and durability of hybridisation with a focus on the influence of (changing) context and practical norms in LMICs.

We therefore contribute to the clinical managerial hybrids literature by describing the important role of practical norms in LMIC healthcare contexts, alongside managerial and professional norms, and ways in which these provide hybrids with a particular form of agency. This is particularly important in Kenya, as in other African country contexts, where people tend to ignore rules, structures and processes and there are few accountability mechanisms (Cleary *et al.*, 2013; Brown, 2016) despite the NPM reforms that have been implemented. We suggest different informal practical norms may also exist in high-income health systems, so the concept may be useful in explaining hybrids’ behaviours there too.

### Practical implications

Understanding how clinician mangers interpret and enact their hybrid roles is vital because of its impact on determining the development of health system’s leadership and governance. Policy makers in developing health systems often attempt to address intractable problems or influence public servants’ behaviours by introducing planning, control or management systems (in line with “new public management” techniques and thinking). However, such reforms have relatively little influence because they fail to address or acknowledge the practical norms affecting behaviours in practice (De Sardan *et al.*, 2017).

Furthermore, public servants are poorly trained to deal with practical and contextual realities of their jobs, including how to deal with practical norms. We suggest that a key skill for clinical managers in LMICs is reading, navigating and when opportune using local practical norms to improve service delivery. Indeed, as de Sardan (2008) notes, at times practical norms may be productive in keeping health systems functioning in circumstances beyond those prescribed by official norms. At other times understanding practical norms may help clinical managers to avoid burning out or losing credibility where making changes is not possible. However, more research is needed to understand the influence of practical norms in often complex LMIC health systems, including when and how they may be used or changed to improve healthcare.

These are skills that few hybrids have learned and the leadership and management training courses offered in LMIC health systems often overlook them. Finally, as in high-income countries (McGivern *et al.*, 2015), it appears that hybrid role models and mentors are crucially important in terms of encouraging Kenyan clinicians and nurses to take on and enact hybrid roles in ways that improve health systems. Hence, rather than providing stand along management and leadership courses, more emphasis needs to be placed on ongoing mentoring and support for junior doctors and nurses consider moving into or at the early stages of enacting hybrid roles.

## Conclusion

We examine how “hybrids”, clinical managers in Kenyan hospitals, interpret and enact their role, framed by competing professional, managerial and practical norms prevalent in LMIC health systems. We empirically extend the analysis of hybrids from developed into LMIC healthcare contexts, where little is known about hybrids, noting the importance of navigating and using practical norms in LMIC healthcare settings, alongside professional and managerial norms. Echoing research in high-income countries, we found some hybrids willing and others reluctant to enact hybrid roles. We make a novel contribution by describing a new type of “ambivalent hybrids”. We explain how ambivalent hybrids both accept and reject hybrid roles at different times, depending on whether prevailing professional, managerial and practical norms enable them to positively influences health services, or simply undermine their agency and professional credibility.

## Figures and Tables

**Table I:** Characteristics of case study hospitals

Characteristic	Hospital A	Hospital B
Estimated annual outpatient visits	129,709	146,924
Estimated annual inpatient admissions	6,665	8,618
Number of beds (and cots)	270	432
Number of staff	300	691
Number of clinical consultants	7	21

## References

[ref001] Anders G. and Makene F.S. (2018), “The importance of practical norms in government health and education services in Tanzania”, British Academy/ Anti-Corruption Evidence Programme Research Paper.

[ref002] Bangura Y. and Larbi G.A. (2006), Public Sector Reform in Developing Countries: Capacity Challenges to Improve Services, Palgrave Macmillan, Basingstoke.

[ref003] Barasa E.W., Molyneux S., English M. and Cleary S. (2017), “Hospitals as complex adaptive systems: a case study of factors influencing priority setting practices at the hospital level in Kenya”, Social Science & Medicine, Vol. 174, pp. 104-112. 28024239 10.1016/j.socscimed.2016.12.026 PMC5267634

[ref004] Blaise P. and Kegels G. (2004), “A realistic approach to the evaluation of the quality management movement in health care systems: a comparison between European and African contexts based on Mintzberg’s organizational models”, The International Journal of Health Planning and Management, Vol. 19 No. 4, pp. 337-364. 15688877 10.1002/hpm.769

[ref005] Brown H. (2016), “Managerial relations in Kenyan health care: empathy and the limits of governmentality”, Journal of the Royal Anthropological Institute, Vol. 22 No. 3, pp. 591-609.

[ref006] Cleary S.M., Molyneux S. and Gilson L. (2013), “Resources, attitudes and culture: an understanding of the factors that influence the functioning of accountability mechanisms in primary health care settings”, BMC Health Services Research, Vol. 13 No. 1, p. 320. 23953492 10.1186/1472-6963-13-320 PMC3844434

[ref007] Corbin J. and Strauss A. (2014), Basics of Qualitative Research: Techniques and Procedures for Developing Grounded Theory, Sage, Los Angeles, CA.

[ref008] Couper I., Hugo J., Ijumba P. and Barron P. (2002), Management of District Hospitals: Suggested Elements for Improvement, Health Systems Trust, Durban.

[ref009] Daire J. and Gilson L. (2014), “Does identity shape leadership and management practice? Experiences of PHC facility managers in Cape Town, South Africa”, Health Policy and Planning, Vol. 29 No. S2, pp. ii82-ii97. 25274644 10.1093/heapol/czu075 PMC4202914

[ref011] De Herdt T. and De Sardan J.-P.O. (2015), Real Governance and Practical Norms in Sub-Saharan Africa: The Game of the Rules, Routledge, Abingdon.

[ref012] De Sardan J.-P. (2015), “Practical norms: informal regulations within public bureaucracies (in Africa and beyond)”, in De Herdt T. and De Sardan J.-P. (Eds), Real Governance and Practical Norms in Sub-Saharan Africa: The Game of the Rules, Routledge, Abingdon.

[ref013] De Sardan J.-P.O., Diarra A. and Moha M. (2017), “Travelling models and the challenge of pragmatic contexts and practical norms: the case of maternal health”, Health Research Policy and Systems, Vol. 15 No. 1, p. 60. 28722553 10.1186/s12961-017-0213-9 PMC5516842

[ref014] Dickinson H., Ham C., Snelling I. and Spurgeon P. (2013), “Are we there yet? Models of medical leadership and their effectiveness: an exploratory study”, final report, NIHR Service Delivery and Organisation Programme, London.

[ref015] Doherty J. (2013), “Strengthening clinical leadership in hospitals”, Municipal Services Project, Cape Town.

[ref016] Doolin B. (2002), “Enterprise discourse, professional identity and the organizational control of hospital clinicians”, Organization Studies, Vol. 23 No. 3, pp. 369-390.

[ref017] Eisenhardt K.M. (1989), “Building theories from case study research”, Academy of Management Review, Vol. 14 No. 4, pp. 532-550.

[ref018] English M., Nzinga J., Mbindyo P., Ayieko P., Irimu G. and Mbaabu L. (2011), “Explaining the effects of a multifaceted intervention to improve inpatient care in rural Kenyan hospitals – interpretation based on retrospective examination of data from participant observation, quantitative and qualitative studies”, Implementation Science, Vol. 6 No. 1, p. 124. 22132875 10.1186/1748-5908-6-124 PMC3248845

[ref019] Ferlie E., Mcgivern G. and Fitzgerald L. (2012), “A new mode of organizing in health care? Governmentality and managed networks in cancer services in England”, Social Science & Medicine, Vol. 74 No. 3, pp. 340-347. 21501913 10.1016/j.socscimed.2011.03.021

[ref020] Fitzgerald L. (1994), “Moving clinicians into management: a professional challenge or threat?”, Journal of Management in Medicine, Vol. 8 No. 6, pp. 32-44. 10161170 10.1108/02689239410073420

[ref021] Fitzgerald L. and Ferlie E. (2000), “Professionals: back to the future?”, Human Relations, Vol. 53 No. 5, pp. 713-739.

[ref022] Fitzgerald L. and Sturt J. (1992), “Clinicians into management: on the change agenda or not?”, Health Services Management Research, Vol. 5 No. 2, pp. 137-147. 10120980 10.1177/095148489200500206

[ref023] Forbes T.O.M. and Hallier J. (2006), “Social identity and self-enactment strategies: adapting to change in professional–manager relationships in the NHS”, Journal of Nursing Management, Vol. 14, pp. 34-42. 16359444 10.1111/j.1365-2934.2005.00614.x

[ref024] Fredrik Bååthe L.E.N. (2013), “Engaging physicians in organisational improvement work”, Journal of Health Organization and Management, Vol. 27 No. I, pp. 479-497. 24003633 10.1108/JHOM-02-2012-0043

[ref025] Freidson E. (2001), Professionalism: The Third Logic, Polity Press, Cambridge.

[ref026] Fulop L. (2012), “Leadership, clinician managers and a thing called ‘hybridity’”, Journal of Health Organization and Management, Vol. 26 No. 5, pp. 578-604. 23115906 10.1108/14777261211256927

[ref027] Gioia D.A., Corley K.G. and Hamilton A.L. (2013), “Seeking qualitative rigor in inductive research”, Organizational Research Methods, Vol. 16 No. 1, pp. 15-31.

[ref028] Goffman E. (1961), Role-Distance, Routledge, London.

[ref029] Goodall A.H. (2011), “Physician–leaders and hospital performance: is there an association?”, Social Science & Medicine, Vol. 73, pp. 535-539. 21802184 10.1016/j.socscimed.2011.06.025

[ref030] Ham C., Clark J., Spurgeon P., Dickinson H. and Armit K. (2011), “Doctors who become chief executives in the NHS: from keen amateurs to skilled professionals”, Journal of the Royal Society of Medicine, Vol. 104 No. 3, pp. 113-119. 21357980 10.1258/jrsm.2011.110042 PMC3046192

[ref031] Hoff T.J. (2000), “Professional commitment among US physician executives in managed care”, Social Science & Medicine, Vol. 50 No. 10, pp. 1433-1444. 10741578 10.1016/s0277-9536(99)00410-4

[ref032] Langley A. and Abdallah C. (2011), “Templates and turns in qualitative studies of strategy and management”, in Bergh D.D. and Ketchen D.J. (Eds), Building Methodological Bridges (Research Methodology in Strategy and Management, Vol. 6), Emerald Group Publishing Limited.

[ref033] Litorpa H., Mgayaa A., Mbekengaa C., Kidantoa H., Johnsdotterc S. and Esséna B. (2015), “Fear, blame and transparency: obstetric caregivers’ rationales for high caesarean section rates in a low-resource setting”, Social Science & Medicine, Vol. 143, pp. 232-240. 26364010 10.1016/j.socscimed.2015.09.003

[ref034] Llewellyn S. (2001), “‘Two-way windows’: clinicians as medical managers”, Organization Studies, Vol. 22, pp. 593-623.

[ref035] McGivern G., Currie G., Ferlie E., Fitzgerald L. and Waring J. (2015), “Hybrid manager–professionals' identity work: the maintenance and hybridization of medical professionalism in managerial contexts”, Public Administration, Vol. 93 No. 2, pp. 412-432.

[ref036] Mintzberg H. (1989), “The structuring of organizations”, Readings in Strategic Management, Palgrave, London.

[ref037] MSH, USAID, MOM Services and MOPH Sanitation (2008), “Report on management and leadership development gaps for Kenya health managers.

[ref038] Noordegraaf M. (2007), “From ‘pure’ to ‘hybrid’ professionalism: present-day professionalism in ambiguous public domains”, Administration & Society, Vol. 39, pp. 761-785.

[ref039] Nyikuri M., Tsofa B., Barasa E., Okoth P. and Molyneux S. (2015), “Crises and resilience at the frontline – public health facility managers under devolution in a sub-county on the Kenyan coast”, PloS One, Vol. 10. 10.1371/journal.pone.0144768 PMC4687913 26696096

[ref040] Nyikuri M.M., Tsofa B., Okoth P., Barasa E.W. and Molyneux S. (2017), “‘We are toothless and hanging, but optimistic’: sub county managers’ experiences of rapid devolution in coastal Kenya”, International Journal for Equity in Health, Vol. 16 No. 1, p. 113. 28911332 10.1186/s12939-017-0607-x PMC5599878

[ref041] Nzinga J., McGivern G. and English E. (2018), “Examining clinical leadership in Kenyan public hospitals through the distributed leadership lens”, Health Policy and Planning, Vol. 33, pp. ii27-ii34. 30053035 10.1093/heapol/czx167 PMC6037084

[ref042] Nzinga J., Mbaabu L. and English M. (2013), “Service delivery in Kenyan district hospitals – what can we learn from literature on mid-level managers?”, Human Resources for Health, Vol. 11 No. 1, p. 10. 23442524 10.1186/1478-4491-11-10 PMC3599555

[ref043] Nzinga J., Mbindyo P., Mbaabu L., Warira A. and English M. (2009), “Documenting the experiences of health workers expected to implement guidelines during an intervention study in Kenyan hospitals”, Implementation Science, Vol. 4 No. 1, p. 44. 19627591 10.1186/1748-5908-4-44 PMC2726115

[ref044] O’Neil M. L. (2008), “Human resource leadership: the key to improved results in health”, Human Resources for Health, Vol. 6 No. 1, p. 10. 18570657 10.1186/1478-4491-6-10 PMC2440377

[ref045] Quinn J.F. and Perelli S. (2016), “First and foremost, physicians: the clinical versus leadership identities of physician leaders”, Journal of Health Organization and Management, Vol. 30 No. 4, pp. 711-728. 27296888 10.1108/JHOM-05-2015-0079

[ref046] Soong C., Wright S.M. and Howell E.E. (2010), “Hospitalist physician leadership skills: perspectives from participants of a leadership conference”, Journal of Hospital Medicine, Vol. 5 No. 3, pp. E1-E4. 10.1002/jhm.637 20235301

[ref047] Tetui M., Hurtig A.-K., Ekirpa-Kiracho E., Kiwanuka S.N. and Coe A.-B. (2016), “Building a competent health manager at district level: a grounded theory study from Eastern Uganda”, BMC Health Services Research, Vol. 16 No. 1, p. 665. 27871333 10.1186/s12913-016-1918-0 PMC5117515

[ref048] The Kenya Health and Leadership Congress, TMOH (2015), The Second National Capacity Assessment Report on Leadership, Management and Governance in Kenya: A National Health Sector Review, Nairobi.

[ref049] Yin R.K. (2013), Case Study Research: Design and Methods, Sage, CA.

